# *Mycobacterium avium* Subspecies *paratuberculosis* in Asymptomatic Zoo Herbivores in Poland

**DOI:** 10.3390/ani13061022

**Published:** 2023-03-10

**Authors:** Małgorzata Bruczyńska, Anna Didkowska, Sylwia Brzezińska, Magdalena Nowak, Katarzyna Filip-Hutsch, Mirosław Kalicki, Ewa Augustynowicz-Kopeć, Krzysztof Anusz

**Affiliations:** 1 Department of Food Hygiene and Public Health Protection, Institute of Veterinary Medicine, Warsaw University of Life Sciences (SGGW), Nowoursynowska 166, 02-787 Warsaw, Poland; 2 County Veterinary Inspectorate, Orezna 9, 05-501 Piaseczno, Poland; 3 Department of Microbiology, National Tuberculosis and Lung Disease Research Institute, 01-138 Warsaw, Poland; 4 Zoological Garden of Gdańsk, Karwieńska 3, 80-328 Gdańsk, Poland

**Keywords:** animals, bongo, herbivore, IS900, *Mycobacterium avium* spp. *paratuberculosis*, one-health, paratuberculosis, zoo

## Abstract

**Simple Summary:**

Paratuberculosis is a bacterial infection occurring globally in ruminants. Although it has a known impact on animal health and welfare, diagnosis is complicated by high animal densities, the chronic nature of the disease, the variable course of infection, and the immune response. The aim of the current study was to confirm whether *Mycobacterium avium* sp. *paratuberculosis* (MAP) infections occur in zoo animals in Poland. Faeces samples (*n* = 131) were collected for analysis from different species of animals from eight zoos in Poland. Our study provides the first confirmation of MAP in bongo antelope and confirms that MAP is present in Polish zoological gardens and requires monitoring, which can be easier now due to new legislation.

**Abstract:**

Mycobacterial infections are significant issues in zoo animals, influencing animal welfare, conservation efforts, and the zoonotic potential of pathogens. Although tuberculosis is recognised to be highly dangerous, paratuberculosis can also lead to animal losses and is potentially dangerous for humans. The aim of the current study was to confirm whether *Mycobacterium avium* spp. *paratuberculosis* (MAP) infections are currently present in zoos in Poland. Faeces samples (*n* = 131) were collected from different animal species from eight zoos in Poland. The faeces were decontaminated and inoculated into Herrold’s Egg Yolk Media. The species was determined using commercial DNA testing. The IS900 was checked using RT-PCR. The culture was positive in seven samples: five with *M. avium*, one with *Mycobacterium fortiatum*, and one without any identified *Mycobacterium* species. RT-PCR confirmed MAP genetic material in nine animals. Our findings represent the first confirmation of MAP in bongo (*Tragelaphus eurycerus*), indicating that it is present in Polish zoological gardens. Fortunately, the disease can be monitored more easily due to recent legislation (the Animal Health Law).

## 1. Introduction

Mycobacterial infections in zoo animals have a significant impact on animal welfare and conservation efforts, and have worrying zoonotic potential [1]. Of these diseases, the most dangerous is believed to be tuberculosis (TB); however, significant animal losses can be caused by paratuberculosis. Paratuberculosis is a chronic granulomatous infectious disease caused by *Mycobacterium avium* subsp. *paratuberculosis* (MAP), an acid-fast bacterium characterised by long environmental persistence. 

The most commonly affected species are ruminants; however, other mammals are also susceptible [2,3]. In zoos, paratuberculosis has been confirmed among *Bovidae* [4,5,6], *Cervidae* [7] *Giraffidae* [8,9], *Camelidae* [10,11], *Rhinocerotidae* [12,13], and *Rodentia* [1,2]. 

In zoos, many animals can be unrecognised reservoirs of MAP; these can have major epidemiological significance by shedding MAP intermittently or chronically [14,15]. Transmission is mostly through the faecal-oral route, although vertical, pseudo-vertical and venereal transmission have been also described [16,17,18]. Animals usually develop clinical signs after a long incubation period. However, it is important to note that MAP can be shed in faeces several months before clinical signs occur. Progressive weight loss, exercise intolerance, and diarrhoea are the main clinical signs observed in clinical paratuberculosis [19].

Although it remains unclear whether MAP is a potential public health threat [20,21,22], visitors to petting zoos and zookeepers should observe safety precautions.

As paratuberculosis can follow a severe course, depending on species and individuals, [1] there is a need to monitor it. This is particularly important in zoos, which are often home to very valuable and endangered species. Although MAP has been confirmed in Poland in livestock [23,24], no studies have yet examined its occurrence in Polish zoos.

In total, 25 zoological gardens are registered in Poland, in 13 regions of the country. Of these, the 11 best examples are members of the European Association of Zoos and Aquariums (EAZA), together with the most important zoos from all over Europe. Only animals born and raised outside the natural environment, and which have no chance of survival otherwise, may be kept and bred in zoos; however, they may also be kept if it is required to protect the population or species, or to achieve scientific goals. In such cases, in accordance with the Animal Health Law (AHL), the animals are subject to the supervision of the competent authority. The aim of the current study was to confirm whether MAP infections occur in zoo animals in Poland.

## 2. Materials and Methods

### 2.1. Material

Faeces were collected from seven Polish zoological gardens: Zoo “A” (*n* = 61), Zoo “B” (*n* = 24), Zoo “C” (*n* = 6), Zoo “D” (*n* = 9), Zoo “E” (*n* = 16), Zoo “F” (*n* = 1), and Zoo “G” (*n* = 9). Samples were also taken from a non-commercial breeding centre “H” (*n* = 5) (Table 1). All tested animals have no symptoms of disease. Non-herbivore species were excluded from the study. Animals showing signs of diarrhoea and emaciation were excluded from the study, because the purpose of the study was to monitor clinically healthy animals. Ethical approval was not required for this study as the samples were collected without any harm to the animals.

The samples were collected from the following animal species: addax antelope (*Addax nasomaculatus*) (*n* = 1), alpaca (*Vicugna pacos*) (*n* = 10), Ankole-Watusi (*Bos taurus*) (*n* = 2), anoa (*Bubalus depressicornis*) (*n* = 2), waterbuck (*Kobus ellipsiprymnus*) (*n* = 1), Bactrian camel (*Camelus bactrianus*) (*n* = 6), Baringo giraffe (*Giraffa camelopardalis rotshildi*) (*n* = 3), capybara (*Hydrochoerus hydrohaeris*) (*n* = 1), Chinese bharal, (*Pseudois nayaur szechuanensis*) (*n* = 1), Chinese goral (*Naemorhedus caudatus arnouxianu*) (*n* = 1), common eland (*Tragepalhus oryx*) (*n* = 9), Djallonké sheep (*Ovis aries*) (*n* = 1), domestic goat (*Capra hircus*) (*n* = 9), domestic yak (*Bos grunniens*) (*n* = 1), dromedary (*Camelus dromedarius*) (*n* = 6), eastern bongo (*Tragelaphus eurycerus isaaci*) (*n* = 11), European bison (*Bison bonasus*) (*n* = 3), European mouflon (*Ovis aries musimon*) (*n* = 2), fallow deer (*Dama dama*) (*n* = 2), giraffe (*Giraffa camelopardalis*) (*n* = 3), guanaco (*Lama guanicoe*) (n = 2), Java mouse-deer (*Tragulus javanicus*) (*n* = 1), llama (*Lama glama*) (*n* = 3), lowland nyala (*Nyala angasii*) (*n* = 1), maned aruis (*Ammotragus lervia*) (*n* = 3), Mesopotamian fallow deer (*Dama mesopotamica*) (*n* = 1), Mishmi takin (*Budorcas taxicolor taxicolor*) (*n* = 1), Nile lechwe (*Kobus megaceros*) (*n* = 1), okapi (*Okapia johnstoni*) (*n* = 2), Père David’s deer (*Elaphurus davidianus*) (*n* = 1), Polish heath sheep (*Ovis orientalis f. aries* “*Wrzosówka*”) (*n* = 2), prairie bison (*Bison bison*) (*n* = 1), pygmy hippopotamus (*Cheoropsis liberiensis*) (*n* = 5), red cow (*Bos taurus*) (*n* = 1), red deer (*Cervus elaphus*) (*n* = 1), Reeves’s muntjac (*Muntiacus reevesi*) (*n* = 2), reticulated giraffe (*Giraffa camelopardalis reticulata*) (*n* = 3), sable antelope (*Hippotragus Niger*) (*n* = 2), scimitar-horned oryx (*Oryx dammach*) (*n* = 1), Shetland pony (*Equus caballus Shetland*) (*n* = 7), Siberian ibex (*Capra sibirica*) (*n* = 2), sika deer (*Cervus nippon dybowskii*) (*n* = 2), sitatunga (*Tragelaphus spekii gratus*) (*n* = 2), South American tapir (*Tapirus terrestris*) (*n* = 2), southern pudu (*Pudu puda*) (*n* = 1), Thorold’s deer (*Cervus albirostris*) (*n* = 1), vicugna (*Vikugna vicugna*) (*n* = 1), Visayan spotted deer (*Rusa alfredi*) (*n* = 2), white-bearded wildebeest (*Connochaetes taurinus albojubatus*) (*n* = 1), and wild goat (*Capra aegagrus*) (*n* = 1). The age of the animals ranged from 5 months to 22 years (average eight years). The material was collected from 48 females and 47 males (for 36 samples, sex could not be determined). The material (131 faecal samples) was collected in two ways: individual samples (*n* = 89) and pulled samples from pens (n = 42).

### 2.2. Culture

The samples were processed by suspension, decontamination, and culture, according to the World Organisation to Animal Health (WOAH) Terrestrial Manual 2021 (https://www.woah.org/en/what-we-do/standards/codes-and-manuals/terrestrial-manual-online-access/, accessed on 15 December 2021). Briefly, 1 g of faeces was transferred to the distilled water and shaken for 30 min at room temperature (RT). The uppermost 5 mL of the faeces suspension was then transferred to a tube containing 20 mL 0.95% 3-Hydroxy-2-phenylcinchoninic acid (HPC). After being inverted several times, the tube was left to stand for 18 h at RT. The undistributed sediment was then inoculated into Herrold’s Egg Yolk Media (HEYM, Becton Dickinson, Franklin Lakes, NJ, US), with and without mycobactin. The media were incubated at 37 °C for eight months and checked for colonies every seven days.

### 2.3. Genetic Analysis

DNA from colonies was isolated using the Genolyse isolation kit (Hain Lifescience, Nehren, Germany).

The strains were classified as non-tuberculosis mycobacteria species using the GenoType Mycobacterium CM test (Hain Lifescience) based on the DNA-Strip technology. Briefly, the DNA was extracted and then subjected to multiplex amplification with biotinylated primers. Following this, reverse hybridisation was conducted.

MAP was detected by real-time PCR using the VetMax *M. paratuberculosis* 2.0 Kit (Thermofisher Scientific, Waltham, MA, USA). The test targets the insertion sequence IS900, part of the IS1110 family of insertion sequences. It was repeated between 14 and 18 times in MAP genome.

All tests were performed according to the manufacturers’ manuals.

## 3. Results

### 3.1. Culture

**Figure 1:**
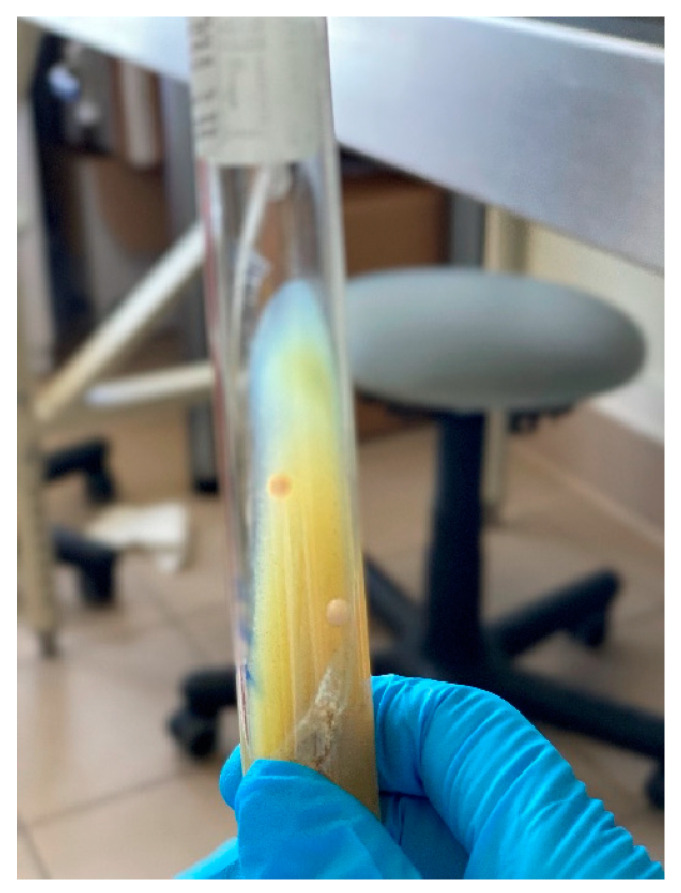
Colonies grown on Herrold’s media with mycobactin.

Positive results were observed in seven samples. Nonchromogenic, small, round, cream-coloured colonies of fastidious cells developed in four to six months on HEYM media with mycobactin (Figure 1).

### 3.2. Genetic Analysis

The genetic analysis confirmed *M. avium* in five isolated strains and *M. fortuitum* in another. One strain was found not to be characteristic of any Mycobacterium species (Table 2). RT-PCR was positive in the case of nine animals from four zoos. Detailed data of animals are presented in Table 3.

## 4. Discussion

Our findings indicate that MAP infections are present in asymptomatic herbivores in Polish zoological gardens. Although not all infected animals develop clinical disease, inflammatory gastrointestinal disease can occur, especially in ruminants [2]. In addition, as asymptomatic infected animals may also be reservoirs of MAP, and hence play a role in its transmission, it is important to confirm the epidemiological status of zoos.

Although infectious diseases are usually monitored using serological methods, in zoos it is difficult to collect sera samples for a large number of animals, so non-invasive materials such as faeces are used. The gold standard diagnostic test in the case of mycobacteria is microbiological culture. While the sensitivity of the test varies according to the type of sample and medium used, it is nevertheless characterised by 100% specificity [25]. In the present study, culture confirmed the presence of *M. avium* in two bongo antelopes originating from Zoo B (Table 1, Table 2 and Table 3), and MAP was confirmed molecularly. While this appears to be the first confirmed case of MAP infection in this species, another bacterium from the *Mycobacterium avium* complex (MAC) has previously been diagnosed in bongo; *M. avium* spp. *hominissuis* (Mah) was confirmed in five captive bongo antelopes suffering from emaciation [26]. Mah was also confirmed in another sitatunga antelope in a Polish zoo [27].

RT-PCR also achieved positive results in the case of seven other species (Table 3). All seven species have previously been confirmed to harbour MAP: pudu [28], guanaco [29,30], European bison [7], giraffe [8], Bactrian camel [31], alpaca [29,32], and domestic goat [33].

In the present study, more positive samples were confirmed by RT-PCR than by culture; nine samples were confirmed molecularly but only two in culture (Table 2 and Table 3). This is a similar result as noted in research on camelids [34]; however, it contrasts with a recent study from a zoo in Mexico [6]. The different sensitivity observed between our diagnostic methods may be due to intermittent excretion or low numbers of bacteria in the faecal sample. Reliable detection of MAP in specific individuals requires repeated, regular sampling. However, as the present study is intended as an epidemiological assessment of the general situation in Polish zoos, samples were only collected once. In addition, some strain types are difficult to cultivate and may have not been detected in culture [35]. In three out of five *M. avium*-positive samples, MAP was not detected by RT-PCR (Table 1 and Table 2). Further tests will be needed to confirm which subspecies has been isolated.

As tuberculosis has previously been confirmed in Polish zoos [36,37], it should be noted that MAP-positive animals can complicate the diagnosis of tuberculosis, due to cross-reactions [38,39,40].

As even asymptomatic animals were found to yield positive results, all zoos should conduct tests in animals showing symptoms that may suggest paratuberculosis. It is important to note that symptoms can vary between ruminants as well as in other species [41]; however, the most common clinical symptom is diarrhoea, leading to wasting and gradual emaciation, while the feed uptake is not affected [42]. As clinical signs of the disease are often inapparent [41], a key tool for controlling paratuberculosis in zoos is necropsy, although gross pathology does not develop in all species [43]. Furthermore, caseation and calcification of lesions have been confirmed in small ruminants, deer, and camelids, which can be mistaken for tuberculosis [44]. In histological examination, paratuberculosis manifests with histiocytic granulomatous inflammation, mucosal thickening, and atrophy of intestinal villi and glands [45].

A key consideration for zoo owners concerns legal action in the case of paratuberculosis being confirmed in a zoo. Since 21 April 2021, within the territory of the Republic of Poland, as in the territories of all other countries belonging to the European Union, Regulation (EU) 2016/429 of the European Parliament and of the Council of 9 March 2016 on transmissible animal diseases and amending and repealing certain acts in the field of animal health (Journal of Laws of the European Union L No. 84, p. 1, as amended) also known as the Animal Health Law (AHL), has been in force. In some areas, the AHL has introduced changes in the field of animal health protection, one of which is the division of infectious animal diseases into five categories (A, B, C, D, E). The AHL regards paratuberculosis as a category E disease, indicating that it requires surveillance in the EU, and that notification, reporting, and surveillance rules apply. The AHL introduces a more universal, but very general, division of all animals into *kept animals*, i.e., those that are kept by humans, and *wild animals*, i.e., those that are not. Zoo animals, being under human control, are regarded as kept animals. Unfortunately, insufficient information exists concerning sick animals in zoos or on private farms to conduct a full epizootic investigation and thus identify the source of paratuberculosis infection [46].

Although the zoonotic potential of MAP remains uncertain [20], it is important to monitor this disease to ensure public health. This is particularly important in zoos, which often have separate areas where children can pet the animals, and where behaviours conducive to faecal–oral infections can often be observed [47].

Based on the distribution of the tested zoological gardens (Table 1), location does not seem to play an important role in the chance of infection. Effective control of MAP infections in zoo animals requires preventive measures, the most important of which is the introduction of strict hygiene measures. In addition, individuals with unknown MAP status should be tested before being introduced to the zoo, and comprehensive pathology and disease monitoring programmes should be adopted [48]. Additionally, as wildlife faeces are known to play an important role as a source of infection for livestock, effective zoo-wide pest control programmes are important [49].

## 5. Conclusions

This study confirms MAP in zoo animals in Poland, and is the first to identify MAP in bongo antelope. Out of 131 samples of asymptomatic animals, genetic analysis confirmed *M. avium* in five isolated strains and *M. fortuitum* in one. Our findings confirm that MAP infections are present in asymptomatic animals in Polish zoological gardens, and that there is a growing need for effective control programmes. All animals with symptoms that may suggest paratuberculosis should be tested for the disease, especially because it is a potential threat to zoo visitors. It is also particularly important that, in line with the requirements of the AHL, disease prevention measures should be included for the exchange or trade in animals. Our study is therefore significant not only because of animal health monitoring, but also for public health protection. It also sets out further possible directions for research in zoos, which should include examinations of animals showing clinical signs typical of paratuberculosis and an attempt to carry out serological monitoring.

## Figures and Tables

**Table 1:** Characteristics of the zoos in which material was collected (A–H).

Code	Area in Hectares	Characteristics of the Place	Number of Visitors per Year 2022	Number of Kept Species/Animals
**A**	over 100	away from the urban agglomeration, in a forested area	500,000–1 mln	156/771
**B**	30.3	isolated from the agglomeration, in an island area	below 50,000	227/1553
**C**	16	forest park, by walking trails	500,000–1 mln	260/1400
**D**	33	near the urban agglomeration, by a river and walking trails	over 1 mln	1132/10,000
**E**	40	near the urban agglomeration	500,000–1 mln	500/12,000
**F**	13.81	near the city centre	below 500,000	312/3547
**G**	16	near park areas	over 1 mln	554/3350
**H**	4	in a forest, private property, agritourism farm	20,000	29/211

**Table 2:** Animals with positive mycobacteria culture results.

ID	Animal Species	Age [Years]	Sex	Zoo	Genetic Analysis
Z24	Bongo *Tragelaphus eurycerus*	5	F	B	*Mycobacterium avium*
Z25	Bongo *Tragelaphus eurycerus*	3	M	B	*Mycobacterium avium*
Z26	Bongo *Tragelaphus eurycerus*	1	F	B	*Mycobacterium avium*
Z27	Bongo *Tragelaphus eurycerus*	1	M	B	*Mycobacterium avium*
Z45	The Java mouse-deer *Tragulus javanicus*	5	M	G	*Mycobacterium avium*
Z106	Red deer *Cervus elaphus*	15	F	C	None of the *Mycobacteria* species
Z194	Sheep *Ovis aries*	11	F	A	*Mycobacterium fortuitum*

**Table 3:** Animals positive for IS900 in RT-PCR.

ID	Animal Species	Age [Years]	Sex	Zoo
Z17	Southern pudu (*Pudu puda*)	2	F	B
Z24	Bongo (*Tragelaphus eurycerus*)	5	F	B
Z25	Bongo (*Tragelaphus eurycerus*)	3	M	B
Z46	Guanaco (*Lama guanicoe*)	11	M	G
Z88	European bison (*Bison bonasus*)	Pulled sample		D
Z113	Giraffe (*Giraffa camelopardalis*)	11	M	A
Z164	Bactrian camel (*Camelus bactrianus*)	5	M	A
Z168	Alpaca (*Vicugna pacos*)	5	F	A
Z192	Domestic goat (*Capra hircus*)	6	F	A

## Data Availability

Not applicable.
